# Brain volumetric alterations accompanied with loss of striatal medium-sized spiny neurons and cortical parvalbumin expressing interneurons in *Brd1*^+/−^ mice

**DOI:** 10.1038/s41598-018-34729-5

**Published:** 2018-11-07

**Authors:** Per Qvist, Simon F. Eskildsen, Brian Hansen, Mohammad Baragji, Steffen Ringgaard, Jolien Roovers, Veerle Paternoster, Simon Molgaard, Thomas Juhl Corydon, Hans Stødkilde-Jørgensen, Simon Glerup, Ole Mors, Gregers Wegener, Jens R. Nyengaard, Anders D. Børglum, Jane H. Christensen

**Affiliations:** 1 iPSYCH, The Lundbeck Foundation Initiative for Integrative Psychiatric Research, Aarhus, Denmark; 2 Department of Biomedicine, Aarhus University, Aarhus, Denmark; 3 iSEQ, Centre for Integrative Sequencing, Aarhus University, Aarhus, Denmark; 4 Center of Functionally Integrative Neuroscience, Department of Clinical Medicine, Aarhus University, Aarhus, Denmark; 5 The MR Research Centre, Department of Clinical Medicine, Aarhus University, Aarhus, Denmark; 6 Department of Ophthalmology, Aarhus University Hospital, Aarhus, Denmark; 7 Psychosis Research Unit, Aarhus University Hospital, Risskov, Denmark; 8 Translational Neuropsychiatry Unit, Aarhus University Hospital, Aarhus, Denmark; 9 Core Center for Molecular Morphology, Section for Stereology and Microscopy, Centre for Stochastic Geometry and Advanced Bioimaging, Department of Clinical Medicine, Aarhus University, Aarhus, Denmark

## Abstract

Schizophrenia is a common and severe mental disorder arising from complex gene-environment interactions affecting brain development and functioning. While a consensus on the neuroanatomical correlates of schizophrenia is emerging, much of its fundamental pathobiology remains unknown. In this study, we explore brain morphometry in mice with genetic susceptibility and phenotypic relevance to schizophrenia (*Brd1*^+/−^ mice) using postmortem 3D MR imaging coupled with histology, immunostaining and regional mRNA marker analysis. In agreement with recent large-scale schizophrenia neuroimaging studies, *Brd1*^+/−^ mice displayed subcortical abnormalities, including volumetric reductions of amygdala and striatum. Interestingly, we demonstrate that structural alteration in striatum correlates with a general loss of striatal neurons, differentially impacting subpopulations of medium-sized spiny neurons and thus potentially striatal output. Akin to parvalbumin interneuron dysfunction in patients, a decline in parvalbumin expression was noted in the developing cortex of *Brd1*^+/−^ mice, mainly driven by neuronal loss within or near cortical layer V, which is rich in corticostriatal projection neurons. Collectively, our study highlights the translational value of the *Brd1*^+/−^ mouse as a pre-clinical tool for schizophrenia research and provides novel insight into its developmental, structural, and cellular pathology.

## Introduction

Schizophrenia (SZ) is a chronic and severe mental disorder that has at its origin structural and functional brain changes^1^. Whereas our understanding of the genetic and environmental predispositions underlying these changes is increasing^2–4^, the neuro-molecular and -cellular defects that contribute to disorder initiation and/or progression are yet poorly understood. Epigenetic regulation of neurodevelopment contributes to the structural and functional shaping of the brain^5^ and has been implicated with the pathophysiology of SZ^6^. The Bromodomain containing 1 gene, (*BRD1*) encodes a scaffold protein which, through its function in complex with histone modifiers and chromatin remodelers^7,8^, controls the expression of a comprehensive chromatin interactome enriched with genes important in neurodevelopment and mental health^8^. In accordance, *BRD1* has been implicated with both brain development^9^ and susceptibility to mental illness^10–12^, including gene-wise significant association in the currently largest SZ genome-wide association study (GWAS) mega-analysis^4^. A SZ case carrying a disruptive *BRD1* mutation has furthermore been identified^13^. We have previously reported behavioral, molecular, and neurochemical changes with overall translational relevance to SZ in genetically modified mice with mono-allelic inactivation of *Brd1* (*Brd1*^+/−^ mice)^14,15^. These include cognitive and social deficits, decrease in cortical parvalbumin immunoreactive interneurons, hypersensitivity to psychotomimetic drugs coupled with monoaminergic dysregulation and differential cortical and striatal expression of genes that are enriched for SZ risk^14,15^. In this research, we investigate the neuropathological impact of reduced *Brd1* expression in mice using postmortem 3D MR imaging coupled with histology, immunostaining and regional mRNA marker analysis, and we relate our data to the most recent clinical findings in SZ.

## Results

### Brain structural changes in *Brd1*^+/−^ mice

**Figure 1:**
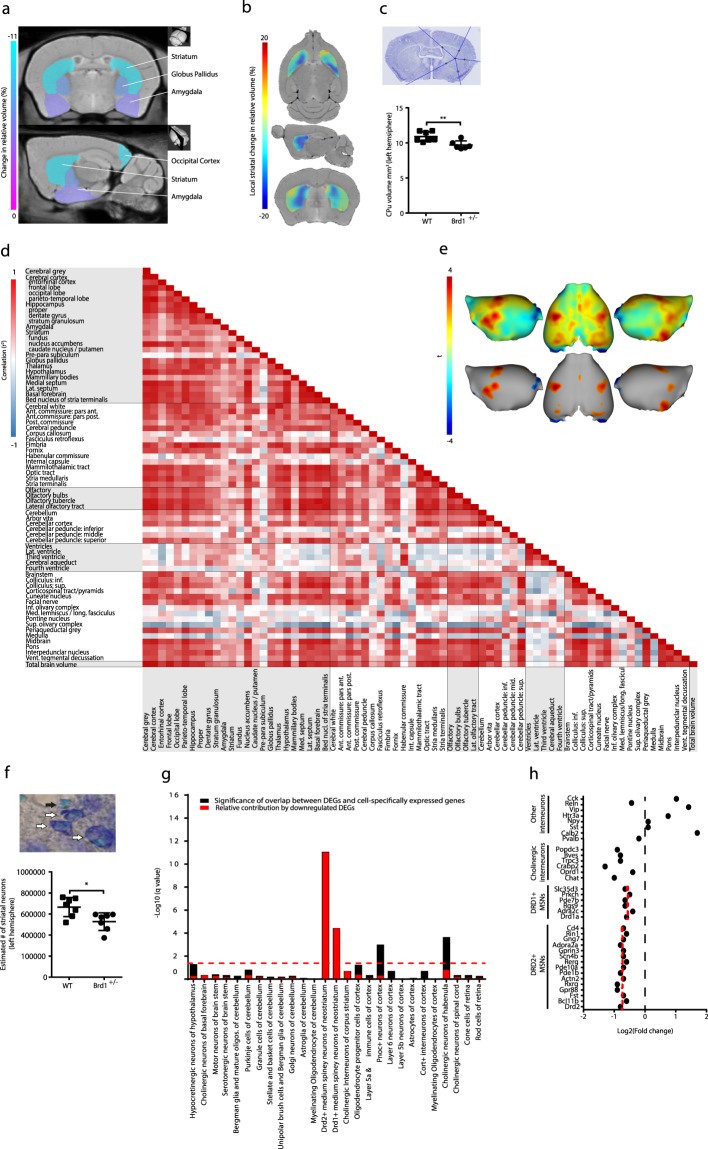
Assessment of brain structural changes using 3D structural magnetic resonance imaging, cell-type specific expression analysis of striatal differentially expressed genes, and estimation of striatal volume and neuron numbers using stereology in *Brd1*^+/−^ and WT mice. (**a**) Coronal and sagittal MRI slices showing the nominally significant differences in absolute volume of subcortical structures between 15 weeks old *Brd1*^+/−^ (n = 10) and WT (n = 9) mice. The percentage change in brain structure volume is indicated by the color bar on the left. (**b**) Horizontal, sagittal and coronal MRI slices showing local deformations between the average of *Brd1*^+/−^ and WT mice within striatum. The percentage voxel-based change in volume is indicated by the color bar on the left. (Red color marks *Brd1*^+/−^ mice larger and blue color marks *Brd1*^+/−^ smaller). (**c**) Stereological estimate on CPu volume in histological samples from an independent batch of 15 weeks old mice, further supports a significant volume loss in *Brd1*^+/−^ (n = 6) compared to WT (n = 7) mice (t test, p = 0.0069). (**d**) A heat map of the correlation coefficient matrix for volumes in all brain regions, structures, and sub-structures in *Brd1*^+/−^ and WT mice. The color scale on the left side shows the strength of the correlation using white (no correlation), blue (negative correlation), and red (positive correlation). (**e**) Illustration of cortical surface showing the localization of groupwise differences in cortical thickness. Only uncorrected p values are presented. Top: t-map (red color marks *Brd1*^+/−^ mice thicker and blue color marks *Brd1*^+/−^ thinner). Bottom: t-map with threshold cutoff at p < 0.05. (**f**) Stereological estimation of total number of CPu neurons show a significant reduction in 15 weeks old *Brd1*^+/−^ (n = 6) compared to WT (n = 7) mice (t test, p = 0.012). White arrows points to neurons, whereas black arrow points to glia. (**g**) Cell specific expression analysis (CSEA) of DEGs identified in dorsal striatum of 8 weeks old *Brd1*^+/−^ mice. Black bars show the Fisher’s Exact Benjamini-Hochberg (BH) corrected p values (−log_10_(q value)) of overlap between DEGs and 27 broad and specific cell type gene sets (at CSEA specificity threshold set to 0.0001). Red bars show the relative contribution of downregulated DEGs to that significance. Enrichments were regarded as statistically significant at q < 0.05 as indicated by the red dotted line. (**h**) Log2 fold change in expression of mRNA markers of various striatal cell types. Red dotted line indicates groupwise mean for markers for D1+ and D2+ MSNs, respectively. The decrease in markers for D2+ MSNs was moderately larger than that for D1+ MSNs (t test, p = 0.008). *p < 0.05; **p < 0.01; ***p < 0.001.

To assess whether reduced *Brd1* expression is associated with gross neuroanatomical changes, we subjected adult *Brd1*^+/−^ (n = 10) and WT (n = 9) mice to high-resolution 3D MR imaging, in combination with deformation-based morphometry analyses. No differences were seen in total grey matter, white matter or overall regional volumes (Table 1). However, *Brd1*^+/−^ mice were characterized by nominally significant volume reductions in several, particular, sub-cortical brain structures. Most significantly, a ~9% volume reduction was observed in striatum (Table 1 and Fig. 1a, p = 0.01), which was mainly driven by a ~10% reduction in the caudate putamen (CPu) (Table 1 and Fig. 1b, p < 0.01). The adjacent globus pallidus was reduced by ~7% (p = 0.03), amygdala by ~5% (p = 0.01), and a tendency for decreased hippocampal volume was observed (p = 0.07). In support of these findings, striatal and amygdaloid volume reductions were observed in both hemispheres independently (CPu: left, p = 0.04; right, p = 0.05; amygdala: left, p = 0.03; right, p = 0.03). Further supporting volume loss, a stereological estimate in fixed tissue from an independent group of mice (7 WT and 6 *Brd1*^+/−^) showed a comparable ~11% total CPu volume reduction in *Brd1*^+/−^ mice compared to WT mice (Fig. 1c, p < 0.01, Table 2). Although total brain volume did not differ between *Brd1*^+/−^ and WT mice (Table 1), a moderate to strong positive correlation was observed between total brain volume and volumes of most regions, structures and substructures excluding the ventricles (Fig. 1d). Furthermore, a positive moderate correlation was observed between ventricular volume and the volume of the hippocampus, amygdala, and CPu – in accordance with all of these structures being reduced in size in *Brd1*^+/−^ mice. On the other hand, a considerable negative correlation was apparent between ventricle-, and CPu volume and corticospinal tract volume (Fig. 1d). In the cerebral cortex, structural alterations were observed in the occipital lobe, where a significant volume reduction was noted in *Brd1*^+/−^ mice compared to WT mice (Table 1, p = 0.02). Assessment of cortical thickness further revealed nominal significant thinning in this region along with thickening of the sensorimotor cortex (Fig. 1e

**Table 1:** 3D MR imaging-based brain volumetric analysis.

Region	Structure	Sub-structure	Average (mm^3^)		Change in absolute volume (%)	Groupwise difference uncorrected p value
			WT	*Brd1* ^+/−^		
Cerebral grey	Cerebral Cortex	Frontal Cortex	46.0 ± 0.9	44.7 ± 0.8	−2.8	0.30
		Occipital Cortex	6.5 ± 0.2	5.9 ± 0.2	−9.8	**0.02**
		Parieto-temporal Cortex	80.3 ± 2.0	78.3 ± 2.2	−2.5	0.51
		Entorhinal Cortex	10.1 ± 0.7	9.6 ± 0.6	−5.2	0.55
			143.0 ± 3.3	138.5 ± 3.6	−3.1	0.37
	Hippocampus	Hippocampus Proper	24.0 ± 0.4	22.7 ± 0.5	−5.5	0.07
		Dentate Gyrus	4.6 ± 0.3	4.1 ± 0.1	−10.8	0.14
		Stratum Granulosum	1.1 ± 0.0	1.0 ± 0.0	−6.0	0.11
			29.7 ± 0.7	27.8 ± 0.7	−6.4	0.07
	Amygdala		16.3 ± 0.2	15.5 ± 0.2	−5.3	**0.01**
	Striatum	Caudate Nucleus/Putamen	20.5 ± 0.5	18.4 ± 0.5	−10.3	**<0.01**
		Fundus of Striatum	0.2 ± 0.0	0.2 ± 0.0	0.9	0.90
		Nucleus Accumbens	4.4 ± 0.2	4.3 ± 0.1	−3.6	0.45
			25.1 ± 0.6	22.8 ± 0.5	−9.1	**0.01**
	Subiculum (pre-para)		0.3 ± 0.1	0.4 ± 0.1	28.9	0.38
	Globus Pallidus		3.2 ± 0.1	3.0 ± 0.1	−6.9	**0.03**
	Thalamus		18.4 ± 0.4	17.6 ± 0.5	−4.1	0.27
	Hypothalamus		12.1 ± 0.3	11.6 ± 0.4	−3.7	0.39
	Mammillary Bodies		0.6 ± 0.0	0.6 ± 0.1	0.1	1.00
	Medial Septum		1.5 ± 0.1	1.4 ± 0.1	−3.0	0.64
	Lateral Septum		3.9 ± 0.2	3.8 ± 0.2	−2.5	0.76
	Basal Forebrain		5.0 ± 0.1	4.9 ± 0.2	−1.8	0.68
	Bed Nucleus of Stria Terminalis		1.5 ± 0.1	1.5 ± 0.1	0.2	0.98
			260.6 ± 5.7	249.5 ± 5.7	−4.3	0.19
Cerebral white	Anterior Commissure: pars anterior		1,7 ± 0.1	1.5 ± 0.0	−9.5	**0.01**
	Anterior Commissure: pars posterior		0,5 ± 0.0	0.5 ± 0.0	−1.9	0.80
	Posterior Commissure		0.1 ± 0.0	0.1 ± 0.0	−4.5	0.43
	Cerebral Peduncle		2.6 ± 0.1	2.5 ± 0.1	−4.3	0.37
	Corpus Callosum		17.1 ± 0.4	16.5 ± 0.2	−3.1	0.18
	Fasciculus Retroflexus		0.3 ± 0.0	0.3 ± 0.0	0.8	0.87
	Fimbria		3.5 ± 0.1	3.2 ± 0.1	−7.4	0.12
	Fornix		0.8 ± 0.1	0.7 ± 0.1	−2.8	0.79
	Habenular Commissure		0.0 ± 0.0	0.0 ± 0.0	−11.0	0.38
	Internal Capsule		3.0 ± 0.1	2.8 ± 0.1	−5.7	0.15
	Mammilothalamic Tract		0.36 ± 0.0	0.3 ± 0.0	−2.1	0.74
	Optic Tract		1.9 ± 0.1	1.9 ± 0.1	−0.4	0.94
	Stria Medullaris		0.8 ± 0.0	0.8 ± 0.0	−2.7	0.62
	Stria Terminalis		1.0 ± 0.0	1.0 ± 0.0	−5.4	0.25
			33.4 ± 0.6	32.1 ± 0.5	−4.1	0.10
Olfactory	Olfactory Bulbs		29.2 ± 0.5	29.4 ± 0.7	0.5	0.88
	Olfactory Tubercle		4.1 ± 0.1	3.9 ± 0.1	−3.4	0.45
	Lateral Olfactory Tract		1.6 ± 0.1	1.6 ± 0.1	−3.3	0.48
			34.9 ± 0.6	34.9 ± 0.9	−0.1	0.96
Cerebellum	Arbor Vita of Cerebellum		9.6 ± 0.3	9.7 ± 0.3	1.1	0.81
	Cerebellar Cortex		47.8 ± 1.4	48.5 ± 1.3	1.5	0.70
	Cerebellar Peduncle: Inferior		1.0 ± 0.1	0.9 ± 0.1	−11.9	0.15
	Cerebellar Peduncle: Middle		1.5 ± 0.1	1.4 ± 0.0	−5.3	0.22
	Cerebellar Peduncle: Superior		0.9 ± 0.1	0.9 ± 0.1	−0.1	0.99
			60.8 ± 1.6	61.4 ± 1.6	1.1	0.78
Ventricles	Lateral Ventricle		4.5 ± 0.2	4.0 ± 0.2	−12.2	**0.04**
	Third Ventricle		1.6 ± 0.1	1.5 ± 0.0	−6.3	0.24
	Cerebral Aqueduct		0.7 ± 0.2	0.6 ± 0.1	−17.7	0.43
	Fourth Ventricle		0.4 ± 0.1	0.3 ± 0.1	−7.1	0.74
			7.2 ± 0.3	6.4 ± 0.3	−11.2	0.06
Brainstem	Colliculus: Inferior		5.7 ± 0.2	5.8 ± 0.3	2.1	0.72
	Colliculus: Superior		9.3 ± 0.5	9.3 ± 0.6	0.8	0.93
	Corticospinal Tract		2.3 ± 0.2	3.3 ± 0.6	41.9	0.14
	Cuneate Nucleus		0.2 ± 0.0	0.2 ± 0.0	1.0	0.94
	Facial Nerve		0.4 ± 0.1	0.3 ± 0.1	−17.2	0.50
	Inferior Olivary Complex		0.6 ± 0.1	0.7 ± 0.1	7.5	0.57
	Medial Lemniscus/Medial Longitudinal Fasciculus		2.5 ± 0.2	2.5 ± 0.1	1.3	0.87
	Pontine Nucleus		0.9 ± 0.0	0.9 ± 0.0	−0.5	0.91
	Superior Olivary Complex		0.8 ± 0.0	0.8 ± 0.0	−8.7	0.18
	Periaqueductal Grey		4.2 ± 0.2	4.2 ± 0.2	−0.7	0.93
	Medulla		25.5 ± 2.5	23.4 ± 1.7	−8.2	0.49
	Midbrain		14.0 ± 0.4	13.8 ± 0.6	−1.4	0.78
	Pons		16.8 ± 0.7	16.2 ± 0.7	−3.6	0.55
	Interpedunclar Nucleus		0.3 ± 0.0	0.3 ± 0.0	−2.1	0.76
	Ventral Tegmental Decussation		0.1 ± 0.0	0.1 ± 0.0	−1.6	0.83
			83.4 ± 2.7	81.6 ± 2.2	−2.2	0.60
Total Brain volume			464.0 ± 9.6	450.4 ± 10.00	−2.9	0.80

3D MR imaging coupled with deformation-based morphometry analyses of differences in absolute cerebral volumes (whole brain, brain regions, brain structures and sub-structures) of 15 weeks old *Brd1*^+/−^ mice (n = 9) compared to WT mice (n = 10). The change in volume was calculated as the percentage change between the mean volume of the WT group and the mean volume of the *Brd1*^+/−^ group. Nominally significant groupwise differences are in bold.

).

**Table 2:** Volumes and cell numbers in WT and *Brd1*^+/−^ mice.

	WT				*Brd1* ^+/−^				p value
	Goup mean	SD	CV	CE^2^/CV^2^	Goup mean	SD	CV	CE^2^/CV^2^	
CPu (µm^3^)	10.90	0.70	0.06		9.70	0.59	0.06		<0.01
CPu (neurons)	666016	90787	0.14	0.16	527479	84378	0.16	0.12	<0.01
aCC (PV+ neurons)	9481	1639	0.17	0.10	6294	1536	0.24	0.05	<0.01
aCC (VVA+ neurons)	9837	1600	0.16	0.11	7206	1334	0.19	0.09	<0.01
aCC (PV+ and neurons)	9375	1633	0.17	0.10	6157	1556	0.25	0.05	<0.01

CPu: cuadate putamen, aCC: anterior cingulate cortex, PV: parvalbumin, VVA: Vicia Villosa Agglutinin, SD: standard deviation, CV: coefficient of variation, CE: coefficient of error.

### Loss of CPu neurons in *Brd1*^+/−^ mice

In order to assess a potential cellular underpinning of the structural change in CPu, we performed a stereological estimate of total CPu neurons in fixed tissue from an independent group of mice (7 WT and 6 *Brd1*^+/−^). In line with reduced CPu volume in *Brd1*^+/−^ mice, a significant (~20%) reduction in striatal neurons was seen in *Brd1*^+/−^ compared WT mice (Fig. 1f, p < 0.01, Table 2). To assess the molecular implications of reduced Brd1 expression on striatal cells, we utilized previously published RNAseq data^15^ from the dorsal striatum of adolescent *Brd1*^+/−^ and WT mice and performed cell specific expression analysis (CSEA). Non-surprisingly, differentially expressed genes (DEGs) were strongly enriched with genes specifically expressed in striatal neurons, namely medium-sized spiny neurons (MSNs) (Fig. 1g, Dopamine receptor 2 expressing (D2+) MSNs: q = 8.54E-12, and Dopamine receptor 1 expressing (D1+) MSNs: q = 3.61E-05). Indicating that cellular loss is present already in adolescent *Brd1*^+/−^ mice, DEGs overlapping the MSN enriched gene lists were exclusively downregulated in *Brd1*^+/−^ mice, suggesting that this subset of DEGs may not reflect altered gene transcription, but rather a change in cell composition. Only neuronal mRNA markers were overrepresented among striatal DEGs whereas glial-, and oligodendrocyte mRNA markers where not. Notably, the decrease in markers for D2+ MSNs was moderately larger than that for D1+ MSNs (Fig. 1h, p < 0.01). Although not significantly enriched in the DEG set, markers for cholinergic, but not GABAergic interneurons, were additionally decreased in *Brd1*^+/−^ mice (Fig. 1h).

### Reduced number of parvalbumin expressing interneurons in *Brd1*^+/−^ mice

**Figure 2:**
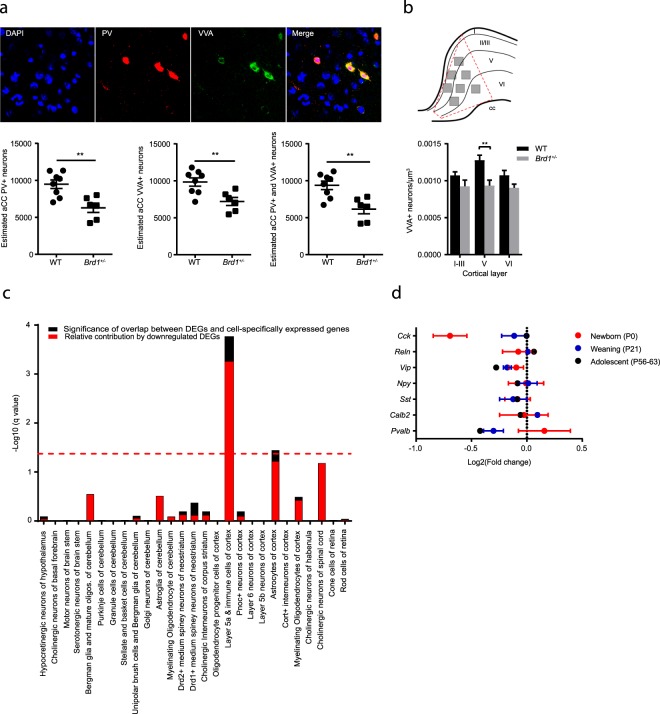
Stereological estimation of difference in numbers of anterior cingulate cortex (aCC) neurons expressing parvalbumin and the lectin, Vicia Villosa Agglutinin, cell-type specific expression analysis of differentially expressed genes detected in aCC, and difference in expression of interneuron markers in cortex at three developmental stages between WT and *Brd1*^+/−^ mice. (**a**) Estimation of aCC neurons stained positive for PV+ and VVA+ in tissue sections from 8 weeks old *Brd1*^+/−^ (n = 6) and WT (n = 8) mice reveals a reduction in cells expressing PV (t test, p = 0.008), VVA (t test, p = 0.003) and both (t test, p = 0.0025) in *Brd1*^+/−^ mice. (**b**) Illustration of image sampling in aCC. 7 image z-stacks were acquired from each hemisphere in 4 different tissue sections per animal. Z-stacks were approximately distributed across cortical layers as illustrated above (3 stacks in layer I-III, 3 in layer V and 1 in layer VI). Reduced number of neurons is restricted to tissue corresponding to cortical layer V (2-way ANOVA, genotype effect, p < 0.001, post hoc test (layer V) p < 0.01). (**c**) Cell specific expression analysis (CSEA) of DEGs identified in aCC of 8 weeks old *Brd1*^+/−^ mice. For further details see legend to Fig. 1g (**d**) Log2 fold change (Log_2_FC) in expression of interneuron markers between WT and *Brd1*^+/−^ mice in whole cortex (newborns (P0), n = 6 in each group, (red)), frontal cortex (juvenile (P21), n = 7 in each group, (blue)), and aCC (adolescent (P56–63), n = 10 in each group, (black)). **p < 0.01.

Although no gross structural abnormalities were identified in the frontal cortex of *Brd1*^+/−^ mice in this study, we have previously reported cortical cellular pathologies, including a significant reduction of parvalbumin immunoreactive (PV+) interneurons in the anterior cingulate cortex (aCC) of *Brd1*^+/−^ mice^15^. Altered levels of interneuron mRNA markers and/or immunoreactivity in the adolescent brain may reflect improper neurodevelopment (e.g., impaired proliferation of neuronal progenitor cells, migration defects or GABA interneuron subtype alteration), neuronal loss by degeneration and/or transcriptional regulation. PV+ interneurons are preferentially ensheathed by perineuronal nets^16,17^ consisting of specialized extracellular matrix components which bind the lectin, Vicia Villosa Agglutinin (VVA). Hence, VVA staining was used as a reliable marker for visualizing the specific neuronal subpopulation of cells potentially expressing PV (Fig. 2a, top). Confirming a reduction of PV+ immunoreactive aCC interneurons, we found a ~30% decrease in PV+ neurons in adolescent *Brd1*^+/−^ compared to WT mice (Fig. 2a, p < 0.01, Table 2). Similarly, we found a ~23% reduction in VVA+ cells (Fig. 2a, p < 0.01, Table 2) and a ~30% decrease in cells expressing both PV and VVA (Fig. 2a. p < 0.01, Table 2), indicating that the reduction in PV+ immunoreactive neurons in adolescent *Brd1*^+/−^ mice is primarily, but not exclusively, due to an absence of neurons. Interestingly, by assessing the regional number of VVA+ neurons in the cortical layers of the aCC, it became apparent that the loss of PV+ interneurons was not equally distributed across aCC tissue, but predominantly localized to the centermost region corresponding to cortical layer V (Fig. 2b, 2-way ANOVA, genotype effect, p < 0.001, post hoc test (layer V) p < 0.01). Further pointing to altered function of neurons in layer V, CSEA on previously published aCC DEGs reported in *Brd1*^+/−^ mice^15^ were overall significantly enriched with markers for layer Va cells (Fig. 2c, q = 1.759e-07), whereas no enrichment was seen in markers for layer Vb or layer VI neurons. mRNA markers for layer Va cells were furthermore predominantly decreased in *Brd1*^+/−^ compared to WT mice (Fig. 2c). To investigate if *Brd1*^+/−^ mice show early signs of interneuron loss, we additionally assessed a selection of cortical interneuron mRNA markers in newborn (P0)-, and juvenile (P21) mice. Surprisingly, in newborn mice, we found a ~40% reduction in *Cck* mRNA (Fig. 2d, p < 0.05), whereas *Cck* mRNA levels were normal in juvenile and adolescent *Brd1*^+/−^ mice. Contrarily, both *Vip* and *Pvalb* mRNA levels were normal in newborn mice and then progressively decreased towards adolescence (Fig. 2d).

## Discussion

*In vivo* neuroimaging has revealed structural brain alterations in SZ cases^1,18–22^ and abnormalities in neuron morphology, density and number has been reported in SZ post-mortem studies^23,24^. Developmental processes linked to genetic, prenatal and environmental factors^25^ as well as post-onset progression^26^ may contribute to these changes. However, medication exposure, patient heterogeneity and the temporal gap between *in vivo* imaging and neuropathological studies complicates the interpretation of these changes in relation to disorder initiation and progression^27^. Here we assess brain morphometry and neuronal mRNA markers in an animal model with construct and face validity to SZ in order to illuminate potential structural- and neuronal pathologies of the disorder.

**Figure 3:**
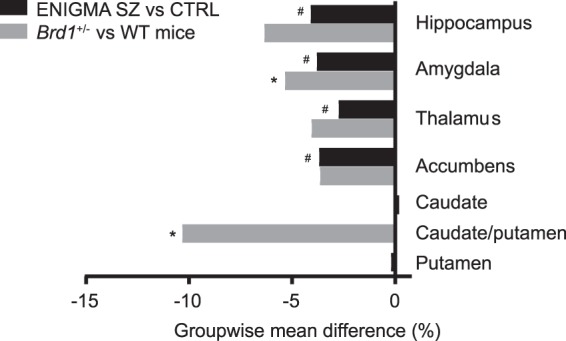
Comparison of group mean difference in volume of subcortical structures in ENIGMA SZ samples and *Brd1*^+/−^ mice. ^#^Marks structures in which mean groupwise volume differ significantly between SZ patients and controls. *Marks structures in which mean groupwise volume differ significantly between *Brd1*^+/−^ and WT mice. In mice, caudate and putamen are not anatomically distinct and are referred to as the caudate-putamen^61^.

*Brd1*^+/−^ mice are characterized by behavioral and neurochemical alterations that implicates widespread brain impairments^15^, including monoaminergic dysregulation and psychotomimetic drug sensitivity. Using an MR imaging based approach, we find volumetric changes in brain tissues that are functionally linked to reported deficits in *Brd1*^+/−^ mice, including volumetric changes in striatum and other subcortical structures. Collectively, our results compare to the findings of the presently largest SZ MRI mega-analysis (2 028 individuals with SZ and 2 540 healthy controls)^1^, which particularly identify subcortical structural abnormalities in SZ (Fig. 3). Importantly, however, whereas SZ cases are characterized by volume reduction of the ventral striatum (accumbens), the striatal volume loss in *Brd1*^+/−^ mice, is seen in the dorsal part (caudate/putamen) (Figs 1b and 3) as has previously been reported in medication free cases^19^. Dorsal striatal activity has been shown to correlate with positive symptoms such as delusions and hallucinations^28^. It serves as the primary gateway of the basal ganglia and play key roles in motor control as well as learning, memory and decision-making^29^, especially in action selection and initiation through the convergence of corticostriatal and thalamostriatal afferents^30^. Noteworthy in this regard, decreased CPu volume correlate negatively with corticospinal tract volume and thickening of the somatosensory cortex in *Brd1*^+/−^ mice. Although there is a large degree of functional homology between the rodent and primate striatum, a direct comparison is complicated by differences in striatal organization^31^, cellular compositions^32^, and efferent connectivity^33^. Striatal volume loss may reflect either neuronal loss, shrinkage of neuron cell bodies and/or non-neural changes, such as loss of glial components. The neuronal types of striatum includes MSNs, which constitute the majority of neurons (>90% in rodents)^34,35^, cholinergic interneurons (1–3%)^36^ and various types of GABAergic interneurons^37^. We find an overall decrease in striatal neurons, with decreased mRNA markers for both MSNs and cholinergic interneurons. MSNs exist as two approximately equally-sized populations based on axonal projections and expression of dopamine receptor subtypes (D2+ or D1+) which have distinctive linkages to intracellular signaling cascades and targets, leading to fundamentally different cellular responses to extracellular dopamine. Namely, activation of dopamine receptor D1 facilitates ATP to cAMP conversion and thus increased cAMP mediated signaling, whereas activation of dopamine receptor D2 inhibits this process^38^. In line with previously reported over-activation of cAMP mediated signaling in the dorsal striatum of *Brd1*^+/−^ mice^15^, we find a larger decrease in D2+ than D1+ mRNA markers. D1+ MSNs project via the direct pathway, and D2+ MSNs via the indirect pathway to the substantia nigra pars reticulate^38^ where they control the activity of afferents to the thalamus, and consequently produce opposing influence on motor output systems^39^. Activation of the direct pathway promotes motor activity whereas stimulation of the indirect pathway inhibits motor activity^40^. The imbalance in striatal D2+/D1+ MSNs suggested in the present study might thus explain the increased sensitivity towards the psychomotor stimulatory effects of cocaine and phencyclidine observed in *Brd1*^+/−^ mice^15^. Striatal cholinergic interneurons, however, differentially modulate MSN excitability dependent on dopamine tone^36^, thus complicating the interpretation. Although the largest structural change was detected in the dorsal striatum, volume reductions were similarly noted in neighboring globus pallidus, and amygdala where the cellular compositions are markedly different from that of striatum (i.e. amygdala is composed of 80% glutamatergic spiny principal neurons and GABA interneurons^41^). Assessment of cellular pathologies in these brain tissues in *Brd1*^+/−^ mice is thus warranted.

Prefrontal cortex (PFC) dysfunction has been implicated in SZ pathology, across symptom domains^42–45^, and alterations of the inhibitory PFC circuitry preferentially involving PV+ interneurons, are among the most robust findings^46^. In agreement with previously reported reduction in the number of PV immunoreactive interneurons in aCC in *Brd1*^+/−^ mice, we find a similar reduction using a different staining method and a different stereological approach. Moreover, we demonstrate the source of reduction primarily lies in reduced number of neurons and not in loss of immunoreactivity. Additionally, the reduction is most prominent in the centermost part of aCC, corresponding to layer V. This is supported by assessment of RNAseq data from the aCC region in *Brd1*^+/−^ mice, showing that DEGs cluster particularly in genes expressed in layer Va. Intriguingly, cortical projections to striatum originates exclusively in cortical layer V^47–49^. Finally, through assessment of interneuron mRNA markers in cortical tissue from newborn and juvenile *Brd1*^+/−^ mice along with RNAseq data from aCC in adolescent mice, we suggest that PV dysfunction might not be restricted to aCC, and that the dysfunction could be progressive and thus, likely neurodevelopmental in its origin. However, further investigations are warranted to delineate the neurodevelopmental impact of reduced Brd1 expression.

Despite recent advances in neuro-genetics, functional neuro-science, and profiling of the brain structural abnormalities in SZ, there has only been limited progress in understanding the pathophysiology and the development of effective therapies. Whereas studies in patients are largely limited to *in vivo* neuroimaging and postmortem examination, the much needed translation of disease pathology into mouse models has proven to be challenging, primarily due to the complex genetic architecture of SZ^50^. In the present study, we assess cellular and structural changes in a well characterized genetically modified mouse model that incorporates SZ genetic risk at three levels: allelic, chromatin interactomic, and brain transcriptomic^15^. We identify volumetric changes in brain structures that are largely overlapping with structural changes reported in the presently largest SZ MR imaging mega-analysis and functionally linked to SZ symptomatology. Intriguingly, we demonstrate that the striatal volume reduction is caused by a general loss of striatal neurons, likely differentially implicating D1+ and D2+ MSNs, thus presumably resulting in overall altered striatal output. Potentially coupled to this defect, we find indication of developmental loss of cortical PV+ neurons in cortical layer V that is rich with corticostriatal projection neurons.

Collectively, our study highlights the translational value of the *Brd1*^+/−^ mouse as a pre-clinical tool for SZ research and provides novel insight into its structural and cellular pathology.

## Methods and Materials

### Animals

A mouse line heterozygous for a targeted deletion in the *Brd1* gene was generated by TaconicArtemis GmbH (Cologne, Germany) on a congenic C57BL/6NTac genetic background as previously described^15^. Mice were group housed 2–8 mice per cage in Macrolon (type II) cages with standard sawdust bedding and standard rodent food and tap water available *ad libitum*. The cages were enriched with igloos, wooden chew blocks and paper for nesting. Mice were kept on a 12:12 hours light–dark cycle and maintained on 23 ± 2 °C temperature. Tail biopsies were collected for Polymerase Chain Reaction (PCR) based genotyping after weaning (P21). Only male mice were used in the study. All studies were carried out in accordance with Danish legislation, and permission for the experiments was granted by the animal welfare committee, appointed by the Danish Ministry of Food, Agriculture and Fisheries – Danish Veterinary and Food Administration.

### 3D structural magnetic resonance imaging (MRI) and deformation-based analysis

Adult male mice (15 weeks old, 9 WT and 10 *Brd1*^+/−^) were carefully euthanized by cervical dislocation and immobilized in the MRI scanner. A Pilot MRI scan showed no structural damage inflicted by this method compared to scans of mice euthanized by lethal injection. Initially, 9 MRI datasets (4 WT and 5 *Brd1*^+/−^) were acquired on a 9.4T Agilent small-bore scanner (Agilent, Santa Clara, CA, USA) using a 20-mm surface coil (RAPID Biomedical Rimpar, Germany) for signal reception. The images were acquired using a 3D fast gradient echo sequence with repetition time (TR) = 13.7 ms, echo time (TE) = 6.9 ms, flip angle = 20°, matrix size = 400 × 400 × 400, field-of-view = 4.0 × 4.0 × 2.0 cm, spatial resolution = 100 × 100 × 50 μm (highest resolution in coronal direction), number of averages = 4, and acquisition time = 2:26 hours.

Following a coil malfunction, the remaining 10 MR datasets were acquired using a Bruker Biospec 9.4T small-bore system equipped with a 25 mm quadrature volume coil. Here, the scan protocol was adjusted to provide similar image quality as obtained with the higher sensitivity surface coil: fast gradient echo, TR = 8 ms, TE = 4 ms, flip angle = 40°, matrix size = 325 × 200 × 256, field-of-view = 2.6 × 1.6 × 1.0 cm, spatial resolution = 80 × 80 × 40 μm (highest resolution in coronal direction), number of averages = 50, and acquisition time = 3:57 hours.

#### Image analysis

MR images were corrected for B1 inhomogeneity using N3^51^ and denoised using a non-local means filter^52^. Brain masks were manually constructed using ITK-SNAP (www.itksnap.org)^53^. Then images were linearly and non-linearly^54^ registered to a neuroanatomical atlas of the C57BL/6 J mouse with 62 different, labelled regions^55^. Region of interest labels were subsequently transformed and resampled to scanner native space using the calculated deformation fields and affine transformations for calculation of individual regional volumes.

#### Cortical thickness analysis

Cortical thickness was calculated as previously described^56^. Briefly, Laplace’s equation, with a fixed boundary condition that differed at the inner and outer surfaces, was solved using the inner and outer surfaces of the cortex defined on the anatomical atlas and transformed to the given mouse. For each point on the cortical surface, the length of a streamline connecting the inside and outside surfaces was used to define the thickness. Cortical thickness was compared between mice on a point-by-point basis using the spatial transformations determined previously by image registration to identify corresponding surface points for each mouse.

### Tissue preparation

For histological analysis of caudate putamen (CPu) tissue, an independent batch of 15 weeks old male mice (7 WT and 6 *Brd1*^+/−^) were used and anterior cingulate cortex (aCC) immunohistochemistry was performed on 8 weeks old mice (8 WT and 6 *Brd1*^+/−^). Mice were deeply anesthetized with isoflurane. Transcardial brain perfusion fixation was initiated using Cardioplex solution followed by 4% formaldehyde via the left ventricle. Brains were dissected and immersed in 4% formaldehyde for 24 hours and then cryoprotected in sucrose solution (30% w/v) for 48 hours followed by snap-freezing. Brains were molded in Tissue-Tek (Sakura, Tokyo, Japan).

### Histology

Brains were sectioned (40 μm, coronally from Bregma +1.94 mm to −2.46 mm) on a Microm HM355 cryostat (Microm International GmbH, Walldorf, Germany) and sections chosen by systematic sampling. Every 6th section was sampled for Toluine Blue staining. Sections were assessed under an Olympus BX50 light microscope equipped with a Prior motorized stage, a Heidenhain microcator, Olympus UPlanSApo 60x oil lens (NA = 1.35) and an Olympus DP70 digital camera controlled by newCAST (Visiopharm, Hoersholm, Denmark) software.

#### Stereological estimate of CPu volume

The 2D nucleator and the Cavalieri estimator were combined for unbiased volume estimation of left CPu in the stained sections using the following equation: V(CPu) = T·∑Area(CPu). T is the distance between sections and Area(CPu) is the summed areas of CPu estimated by the 2D nucleator.

#### Stereological estimate of CPu neurons

The total number of CPu neurons was estimated by the optical fractionator. The step lengths in the x- and y-direction were 400 µm, and the area of the two-dimensional unbiased counting frame was 2 000 µm^2^. Disector height and location was decided by the z-axis analysis and counts were performed between −5 to −15 µm in the z-direction. CPu neurons were identified among toluine blue outlined cells by their characteristic large cytoplasm, large nucleus and prominent nucleolus. The total number of neurons, N(neu), in the CPu was estimated using the optical fractionator:

$${\rm{N}}({\rm{neu}})=(1/{\rm{ssf}})\cdot (1/{\rm{asf}})\cdot (1/{\rm{hsf}})\cdot \sum {{\rm{Q}}}^{-}({\rm{neu}}),$$

where ssf, asf and hsf are referred to as section sampling sample fraction, area sampling fraction and height sampling fraction, respectively.

### Cell-type specific expression analysis and subtype mRNA marker analysis

Cell type–specific enrichment for nominally significant differentially expressed genes (DEGs) previously reported in RNAseq studies on tissue micropunches from dorsal striatum and aCC in 8 weeks old *Brd1*^+/−^ mice^15^ was performed using the cell specific expression analysis (CSEA) tool (http://genetics.wustl.edu/jdlab/csea-tool-2/). This tool contains cell type–specifically expressed genes derived from a translational profiling approach that have identified murine transcriptomes from specific, marker-defined cellular subpopulations^57^. We assessed each set of DEGs (dorsal striatum and aCC) for enrichment of 27 broad and specific cell type gene sets (at CSEA specificity threshold set to 0.0001). Enrichments were regarded as statistically significant at Benjamini-Hochberg corrected p value (q value) < 0.05.

### Immunohistochemistry

Brains were sectioned (40 μm, coronally from Bregma 1.5 mm to 0.5 mm) as described above. Sections were washed (3 × 10 min) in TBS containing 0.3% Triton X-100 (TBS buffer), treated and incubated with target retrieval solution (Dako, Glostrup, Denmark) for 20 minutes at 80 °C and washed again (5 × 15 min). Sections were rinsed and blocked with Donkey anti-mouse Fab fragments (1:10) (Jackson ImmunoResearch, West Grove, USA) for 2 hours followed by 5 days of incubation with primary antibody Oyster 550-labeled (Synaptic Systems, Göttingen, Germany) directed towards parvalbumin (PV). Sections were then incubated with 488-conjugated Vicia Villosa Agglutinin (VVA) lectin (Vector Laboratories, Burlingame, USA) and washed (6 × 10 min) in TBS buffer and (1 × 10 min) in 4′,6-diamidino-2-phenylindole (DAPI) (Sigma-Aldrich, St. Louis, Missouri, USA). Finally, the sections were mounted on glass slides and coverslipped with Prolong Diamond mounting media (Thermofisher Scientific, Waltham, USA). The quality of staining of PV+ and VVA+ interneurons was evaluated using Zeiss LSM710/780 laser-scanning confocal microscopes controlled by ZEN 2009/2011 software (Carl Zeiss, Jena, Germany), respectively, as previously described^58^. 3-dimensional confocal images were taken by collecting stack images (135 μm × 135 μm) with a z-plane step size of 1.5 μm throughout the section depth giving a total of 14–16 stacks per image. A total of 14 z-stacks were acquired per brain section (7 z-stack per hemisphere distributed across cortical layer I-IV, V and VI (see Fig. 2b) with 4 brain sections included per animal. The VVA+ and PV+ cells were counted independently and combined according to the following criteria: (1) Well visible DAPI-stained nucleus; (2) PV staining surrounding the DAPI-stained nucleus for PV+ cells (3) VVA staining surrounding the cell body. The total number of PV+, VVA+ and PV+/VVA+ neurons, N(cells, aCC), in the aCC was estimated using the equation:

$${\rm{N}}({\rm{cells}},{\rm{aCC}})=\frac{\sum {{\rm{Q}}}^{-}({\rm{cells}})}{\sum {\rm{v}}({\rm{dis}})}\cdot {\rm{V}}({\rm{aCC}}),$$

where Q^−^(cells) is the number of cells counted in optical disector, v(dis) is volume of disectors and V(aCC) is total volume of aCC.

#### Quantitative reverse transcription-PCR

Whole cortices were sampled from newborn (P0) (7 WT and 7 *Brd1*^+/−^) and frontal cortex from juvenile (P21) (6 WT and 6 *Brd1*^+/−^) male mice by free-hand dissection. An overview of RNA extraction, quality control, cDNA synthesis, and qPCR protocols can be found in Table S1. Primers were designed to span exon boundaries to avoid amplification of potential contaminating genomic DNA (Table S2). Relative gene expression was calculated by the “standard curve method”^59^ using serial dilutions of a pool of cDNA form all samples as a standard. The expression stability of reference genes (n = 4) within and between experimental groups was determined by analyzing their relative mRNA levels with the Normfinder software^60^. mRNA levels were normalized to the geometric mean of the mRNA levels of the two genes selected as the best combination for normalization.

#### Statistical analysis

MRI, stereological and mRNA data were analyzed using the GraphPad Prism software (San Diego, USA) and Microsoft Excel 2016 (Redmond, Washington, USA9. Data were checked for normal distribution by the Shapiro-Wilk normality test. The statistical significance of group wise comparisons were determined by the student’s *t-test*. Correlation coefficients were calculated using the Correlation data analysis tool in the Microsoft Excel Real Statistics add-on package. Differences in cortical thickness were assessed at each point on the atlas cortical surface using two-sample t-tests. Only uncorrected p values are presented. The resulting maps were thresholded at p < 0.05. For assessment of neuronal loss across cortical layers, a two-way ANOVA with genotype and layer as factors. Tukey’s test was performed as post hoc test and Bonferroni’s corrected p value reported. Data were presented as mean ± SEM, and p values < 0.05 were considered to be statistically significant. Details on the statistic applied in CSEA can be found in (2), where the method is described in detail. Briefly, the overlap of genes identified as differentially expressed between *Brd1*^+/−^ and WT mice and genes highly enriched in certain cell types was compared and a p value for Fisher’s Exact Test is provided. Only cell type specific genes determined with the highest stringencies for enrichment (PSi = 0.0001) were used in this study and only Benjamini Hochberg corrected p-value (q value) < 0.05 were considered significant.

## Electronic supplementary material

Supplementary tables
