# Supplementary material for: Brain volumetric alterations accompanied with loss of striatal medium-sized spiny neurons and cortical parvalbumin expressing interneurons in Brd1+/− mice
Source: Sci Rep. 2018 Nov 7;8:16486. doi: 10.1038/s41598-018-34729-5 (PMC6220279; doi:10.1038/s41598-018-34729-5)
Supplement: Supplementary file 1 — Supplementary tables [file 41598_2018_34729_MOESM1_ESM.docx]

**Supplementary Information**

**Title:** Brain volumetric alterations accompanied with loss of striatal medium-sized spiny neurons and cortical parvalbumin expressing interneurons in *Brd1*^+/-^ mice

**Authors and affiliated institutions:**

Per Qvist^1-3^, Simon F. Eskildsen^4^, Brian Hansen^4^, Mohammad Baragji^2^, Steffen Ringgaard^5^, Jolien Roovers^2^, Veerle Paternoster^1-3^, Simon Molgaard^2^, Thomas J. Corydon^2,6^, Hans Stødkilde-Jørgensen^5^, Simon Glerup^2^, Ole Mors^1,3,7^, Gregers Wegener^8^, Jens R. Nyengaard^9^, Anders D. Børglum^1-3,7^ and Jane H. Christensen^1-3^

1) iPSYCH, The Lundbeck Foundation Initiative for Integrative Psychiatric Research, Aarhus, Denmark 2) Department of Biomedicine, Aarhus University, Aarhus, Denmark 3) iSEQ, Centre for Integrative Sequencing, Aarhus University, Aarhus, Denmark 4) Center of Functionally Integrative Neuroscience, Department of Clinical Medicine, Aarhus University, Aarhus, Denmark 5) The MR Research Centre, Department of Clinical Medicine, Aarhus University, Aarhus, Denmark 6) Department of Ophthalmology, Aarhus University Hospital, Aarhus, Denmark 7) Psychosis Research unit, Aarhus University Hospital, Risskov, Denmark 8) Translational Neuropsychiatry Unit, Aarhus University Hospital, Aarhus, Denmark 9) Core Center for Molecular Morphology, Section for Stereology and Microscopy, Centre for Stochastic Geometry and Advanced Bioimaging, Department of Clinical Medicine, Aarhus University, Aarhus, Denmark

**Table S1** | Overview of RNA extraction, quality control, cDNA synthesis, and qPCR protocols

|  | **Method** | **Quality control** |
| --- | --- | --- |
| **Total RNA isolation** | Newborn: Maxwell® 16 LEV SimplyRNA Tissue kit (Promega Corporation, Madison, USA) Juvenile: AllPrep DNA/RNA Mini Kit (Qiagen, Hilden, Germany) |  |
| **RNA concentration, integrity, and purity** | NanoDrop 1000 version 3.7.1 (Thermo Fisher Scientific) | Criteria: A260/280 O.D. ratio > 1.8 |
|  | 1% agarose gel electrophoresis | Criteria: Presence of 28S and 18S rRNA bands in an approximated 2:1 ratio, no visible degradation, and no visible genomic DNA contamination |
| **cDNA synthesis** | iScript™ cDNA Synthesis Kit (Bio-Rad, Hercules, California, USA) using 1 μg of RNA template. |  |
| **Relative quantification by real-time PCR** | LightCycler®480 System (Roche Applied Science)  SYBR Green chemistry (Bio-Rad Laboratories)  Newborn: 1:20 cDNA dilution, serial dilutions: 1:8, 1:16, 1:32, 1:64 and 1:128  Juvenile: 1:40 cDNA dilution, serial dilutions: 1:5, 1:25, 1:125 and 1:625  All samples were run in triplicates. | Criteria primer efficiency 1.8 - 2.1  Negative controls (cDNA synthesis without reverse transcriptase) included for all samples  Additional melting curve analysis to assess specificity of the primers (68 °C to 97 °C in 1’) |

**Table S2 |** Primers used in this study

| **Gene name** | **Forward (5’-3’)** | **Reverse (5’-3’)** |
| --- | --- | --- |
| \| Aryl hydrocarbon receptor interacting protein gene *(Aip)* \|  \|  \| \| --- \| --- \| --- \| | GCCCAGTTCCTCTGTGACAT | CAGCATCTCGATGTGGAAG |
| \| Phosphoglycerate kinase 1 (*Pgk1*) \|  \| \| --- \| --- \| | GGTGGAATGGCCTTTACCTT | \| GCCAGTCTTGGCATTCTCAT \| \| --- \| |
| \| Hydroxymethylbilane synthase (*Hmbs*) \|  \|  \| \| --- \| --- \| --- \| | TCCCTGAAGGATGTGCCTA | AAGGGTTTTCCCGTTTGC |
| \| Hypoxanthine-guanine phosphoribosyl transferase (*Hprt*) \|  \|  \| \| --- \| --- \| --- \| | CTTTGCTGACCTGCTGGATT | CAACAATCAAGACATTCTTTCCA |
| \| Calretinin *(Calb2)* \|  \|  \| \| --- \| --- \| --- \| | CTCCTGAAAGAAGGCC | GGCGTCCAGTTCATT |
| Cholecystokinin (Cck) | TGCCCTCAACTTAGCTGG | AGCTTCTGCAGGGACTAC |
| Parvalbumin (Pvalb) | AAAAAGAACCCGGAT | GCCAGAAGCGTCTTT |
| \| Reelin *(Reln)* \|  \|  \| \| --- \| --- \| --- \| | ACGTCCTTTCTGGACAGCTC | GCCACCATCTGAACTGGATT |
| \| Somatostatin *(SSt)* \|  \|  \| \| --- \| --- \| --- \| | GAGGCAAGGAAGATG | GGGCATCATTCTCTG |
| Neuropeptide Y (*Npy*) | AGGCTTGAAGACCCTTCCAT | ACAGGCAGACTGGTTTCAGG |
| Vasoactive Intestinal Peptide (*Vip*) | GAAATACCTGAACTCCATCCTGA | TTCTCCAGCTCTTCAAGAAAGTC |
